# Colon capsule endoscopy in patients with chronic respiratory disease during the COVID‐19 pandemic

**DOI:** 10.1111/codi.15443

**Published:** 2020-12-28

**Authors:** Konosuke Nakaji

**Affiliations:** ^1^ Endoscopy Center Aishinkai Nakae Hospital Wakayama Japan

*Dear Editor*,

We read with interest the article by MacLeod et al. [1]. The authors suggest that colon capsule endoscopy (CCE) could be a useful tool for triaging emergency colonoscopy during the COVID‐19 pandemic. The literature for CCE in patients with chronic respiratory disease, who comprise one of the COVID‐19 high‐risk groups [2], is limited. We carried out a retrospective study of CCE in patients with chronic respiratory disease up to May 2020, the first wave of the COVID‐19 pandemic in Japan. Thirty‐one patients (18 men and 13 women with a mean age of 65.2 ± 15.1 years) with chronic respiratory disease who underwent CCE at our hospital were included. The diagnoses of these patients were pulmonary emphysema in seven patients, bronchial asthma in seven patients, chronic bronchitis in six patients, sleep apnoea syndrome in two patients, pulmonary tuberculosis (old and latent) in eight patients and nontuberculous mycobacteriosis in one patient. The reasons for the procedure were as follows: screening in seven patients, positive for a faecal occult blood test in four patients, lower abdominal pain in five patients, diarrhoea in two patients, abnormal bowel movement in five patients, narrowing of the stool column in one patient, follow‐up for Crohn's disease in one patient, suspicion of irritable bowel syndrome in three patients and accumulation of positron emission tomography in the transverse colon in one patient. The overall total colorectal detection rate using CCE was 90.3% (28/31). Polyps were detected in 54.8% of patients by CCE (17/31 cases), and a significant finding (polyp lesion of 6 mm or more) was observed in 64.7% (11/17 cases). Colonoscopy (CS) was performed in 45.1% (14/31). The positive predictive value was 100% (17/17 patients) and the negative predictive value (13/14 patients) was 92.9%. Histopathological examination by therapeutic endoscopy using CS with personal protective equipment revealed six cases of adenoma, six cases of hyperplasia, one case of carcinoma *in situ* and no advanced cancer. There were no adverse events, such as retention, in any patient and no evidence of SARS‐Cov‐2 infection in healthcare professionals. There is a report suggesting the possibility of colon CT examination [3] and positron emission tomography [4] as an alternative method during the COVID‐19 pandemic. However, CCE is free from radiation exposure and the only examination that can be performed at home without coming to hospital during the pandemic. CCE is expensive, it costs the national health insurance adaptation of Japan approximately ￥30 000 at a 30% burden. However, it can be easily inferred that in patients with chronic respiratory disease it is more cost‐effective than the medical cost of ventilators and extracorporeal membrane oxygenation in intensive care units if they become seriously ill with SARS‐Cov‐2 infection. Also, in Japan, CCE was adopted by the national health insurance plan for chronic obstructive pulmonary disease from March 2020. Our study results suggest that CCE in patients with chronic respiratory disease avoids unnecessary CS and may reduce the risk of SARS‐Cov‐2 infection by CS in approximately 60% of the patients.

## CONSENT

All patients consented to data collection, analysis and dissemination.

## AUTHOR CONTRIBUTIONS

The author participated in the interpretation of study results, and in the drafting, critical revision, and approval of the final version of the manuscript.

## CONFLICT OF INTEREST

I declare no conflict of interests.

## ETHICAL STATEMENT

This study was approved by the ethics committee.

## Data Availability

The data that support the findings of this study are openly available in [repository name e.g. “figshare”] at 10.6084/m9.figshare.13278059.
